# British Balneological and Climatological Society

**Published:** 1896-01-25

**Authors:** 


### British Balneological and Climatological Society.

At a meeting of medical practitioners connected with British health resorts, held at Limmer's Hotel, Conduit Street, W., towards the end of last year, the above society was formed, to carry out the following objects :—

To provide means for the Association of Medical Practitioners connected with British Health Resorts, and to promote good fellowship amongst themselves and with members of other branches of the medical profession.

To encourage the theoretical and practical investigation and study of balneo-therapeutics and medical climatology.

To advocate and sustain within the profession the interests and claims of British balneology and climatology.

It had long been felt that such an association was needed, and the recent publication of the first report of the excellent work done by the committee appointed by the Royal Medical and Chirurgical Society to inquire into the balneology and climatology of Great Britain seemed to come partly as a reproach and partly as an incentive to those practitioners at health resorts who are so intimately and practically interested in the subjects. It is hoped that the new society may accomplish much good scientific work and thus assist and further the objects of that committee, as well as secure for British health resorts the recognition and support which they deserve from the profession at large.

All qualified medical practitioners connected with British health resorts are eligible for membership; but although the society is intended primarily and principally for members of that particular branch of the profession, medical men not connected with health resorts, but who take an interest in the subjects of balneology and climatology, are also eligible for membership.

It is proposed to hold most of the meetings for papers, discussions, &c., in London, as being a convenient centre, and the use of a room at Limmer's Hotel has been temporarily secured. Dates and hours will be arranged, as far as possible, to suit the wishes of members. The advisability of holding an annual meeting at a health resort will also be considered.

The inaugural meeting and conversazione was held on Wednesday, January 22nd, at Limmer's Hotel, Conduit Street, W., when addresses were given by Dr. William Miller Ord and others.

The annual subscription has been fixed at 10s. 6d.

Forms of application for membership and any information will be supplied to medical men desirous of joining the society upon application to one of the hon. secretaries.

The following gentlemen have been elected officers of the society :—

Honorary president: Sir Edward H. Sieveking, M.D., F.R.C.P., K.B. Honorary vice-presidents: Sir John Banks, M.D., LL.D., K.C.B.; Edward Robert Bickersteth, F.R.C.S.; J. Mitchell Bruce, M.D., F.R.C.P.; Professor John W. Byers, M.D., M.A. Professor M. Charteris, M.D.; Sir B. Walter Foster, M.D., F.R.C.P., D.C.L., M.P.; W. Miller Ord, M.D., F.R.C.P.; D. Lloyd Roberts, M.D., F.R.C.P., F.R.S.Ed.; Sir Thomas Grainger Stewart, M.D., F.R.C.P., F.R.S.Ed.; Hermann Weber, M.D., F.R.C.P.; C. Theodore Williams, M.D., F.R.C.P.; I. Burney Yeo, M.D., F.R.C.P. President: Henry Lewis, M.D., Folkestone; Vice-presidents: R. Eardley-Wilmot, M.B., Leamington; D. Edgar Flinn, F.R.C.S., M.R.C.P., Kingstown; R. Fortescue Fox, M.D., M.R.C.P., Strathpeffer Spa, N.B.; H. R. Freeman, F.R.C.S., J.P., Bath; William Haldane, M.D., Bridge of Allan; Alfred Haviland, M.R.C.S., L.S.A., Douglas; Samuel Hyde, M.D., Buxton; John Inglis, M.A., M.S., Hastings; Edward Mackey, M.D., M.R.C.P., Brighton; A. S. Myrtle, M.D., J.P., Harrogate; William V. Snow, M.D., M.R.C.P., Bournemouth; George S. Watson, M.R.C.S., L.S.A., Tunbridge Wells. Council: Edwin Baily, M.B., C.M., Oban; George B. Barron, M.D., Southport; R. O. Gifford Bennet, M.D., J.P., Buxton; Andrew A. Brockatt, M.D., Malvern; W. King Bullmore, M.D., Falmouth; C. A. Cardew, M.R.C.S., L.S.A., Cheltenham; J. Michell Clarke, M.D., M.R.C.P., Clifton; H. Evelyn Crook, M.D., F.F.C.S., Margate; R. Cuffe, M.R.C.S., L.S.A., Woodhall Spa; Morgan Dockrell, M.A., M.D., London; Thomas Johnstone, M.D., M.R.C.P., Ilkley; H. Shirley Jones, M.R.C.S., L.S.A., Droitwich; Arthur Kempe, M.D., M.R.C.P., Exmouth; George C. Kingsbury, M.A., M.D., Blackpool; A. Eyton Lloyd, M.D., J.P., Rhyl; Thomas Macqueen, M.B., C.M., Eastbourne; E. F. Martin, M.B., Weston-super-Mare; Henry McClure, M.D., Cromer; William Moxon, M.D., L.R.C.P., Matlock; J. Ivor Murray, M.D., F.R.S., Ed., J.P., Scarborough; James Nicol, M.D., J.P., Llandudno; A. W. Orwin, M.D., M.R.C.P., Ed., London; John Ormsby, M.D., L.R.C.P., Dover; Frederick Pearse, M.D., F.R.C.S., Southsea; William H. Pearse, M.D., M.R.C.P., Plymouth; Reginald Pollard, M.B., Torquay; William A. Shann, M.B., Lowestoft; C. H. Tamplin, M.R.C.S., L.R.C.P., Ramsgate; Alexander Thom, M.A., M.D., Crieff; C. W. E. Toller, M.D., Ilfracombe. Chairman of council: Samuel Hyde, M.D., Buxton. Treasurer: C. R. B. Keetley, F.R.C.S., London. Hon. secretaries: Aylmer A. Macfarlane, L.R.C.P., Brighton; Septimus Sunderland, M.D., London.

